# Hydroclimate change in the Garhwal Himalaya, India at 4200 yr BP coincident with the contraction of the Indus civilization

**DOI:** 10.1038/s41598-021-02496-5

**Published:** 2021-11-29

**Authors:** E. A. Niederman, D. F. Porinchu, B. S. Kotlia

**Affiliations:** 1 Department of Geography, University of Georgia, Athens, GA 30602 USA; 2 Centre of Advanced Study in Geology, Kumaun University, Nainital, Uttarakhand India

**Keywords:** Climate change, Palaeoclimate

## Abstract

High-resolution analysis of a 3.80 m sediment core recovered from Deoria Tal, a mid-elevation lake located at 2393 m a.s.l. in the Garhwal Himalaya, documents long-term and abrupt hydroclimate fluctuations in northern India during the mid- to late Holocene. The sediment chronology, based on ten ^14^C dates, indicates the core spans 5200 years. Non-destructive, radiological imaging approaches (X-ray fluorescence (XRF), X-ray imaging, and CT scans) were used to assess the response of the lake system to changing hydroclimatic conditions. Variations in elemental concentrations and sediment density evidenced notable hydroclimate change episodes centered at 4850, 4200, and 3100 cal yr BP. Elevated detrital input, greater sediment density, decreased lake ventilation, and lower autochthonous productivity reflects lake deepening between 4350 and 4200 cal yr BP. An abrupt shift in elemental concentrations and sediment density indicated the onset of lake drawdown at 4200 cal yr BP and a negative hydroclimate anomaly between 4200 and 4050 cal yr BP. Lower detrital flux, decreased sediment density, increased oxygenation, and higher autochthonous productivity, reflects a reduction in lake volume between 3200 and 3100 cal yr BP. The potential link between abrupt climate change at 4200 cal yr BP and the contraction of the Indus civilization is explored.

## Introduction

The 4200 cal yr Before Present (BP) megadrought, which extended from 4200 to 3900 cal yr BP, was characterized by a reduction in the global monsoon and associated circulation systems, leading to droughts and seasonal precipitation failures worldwide^1^. This event, which included a reduction in the strength of the Mediterranean Westerlies^2^ and a widespread drought over much of the interior of North America^3^, also impacted Asia^4–6^. The East Asian Summer Monsoon (EASM) and the Indian Summer Monsoon (ISM) experienced periods of weakening in eastern China, Inner Mongolia, and India, “synchronous” with the 4200 cal yr BP event^1^. Given that the EASM and the ISM are the main sources of precipitation for their respective regions, climate scientists and archaeologists have postulated that a dramatic reduction in the strength of the Asian summer monsoon at 4200 cal yr BP would have impacted the complex societies present in India and China during the mid- to late Holocene^7,8^.

**Figure 1:**
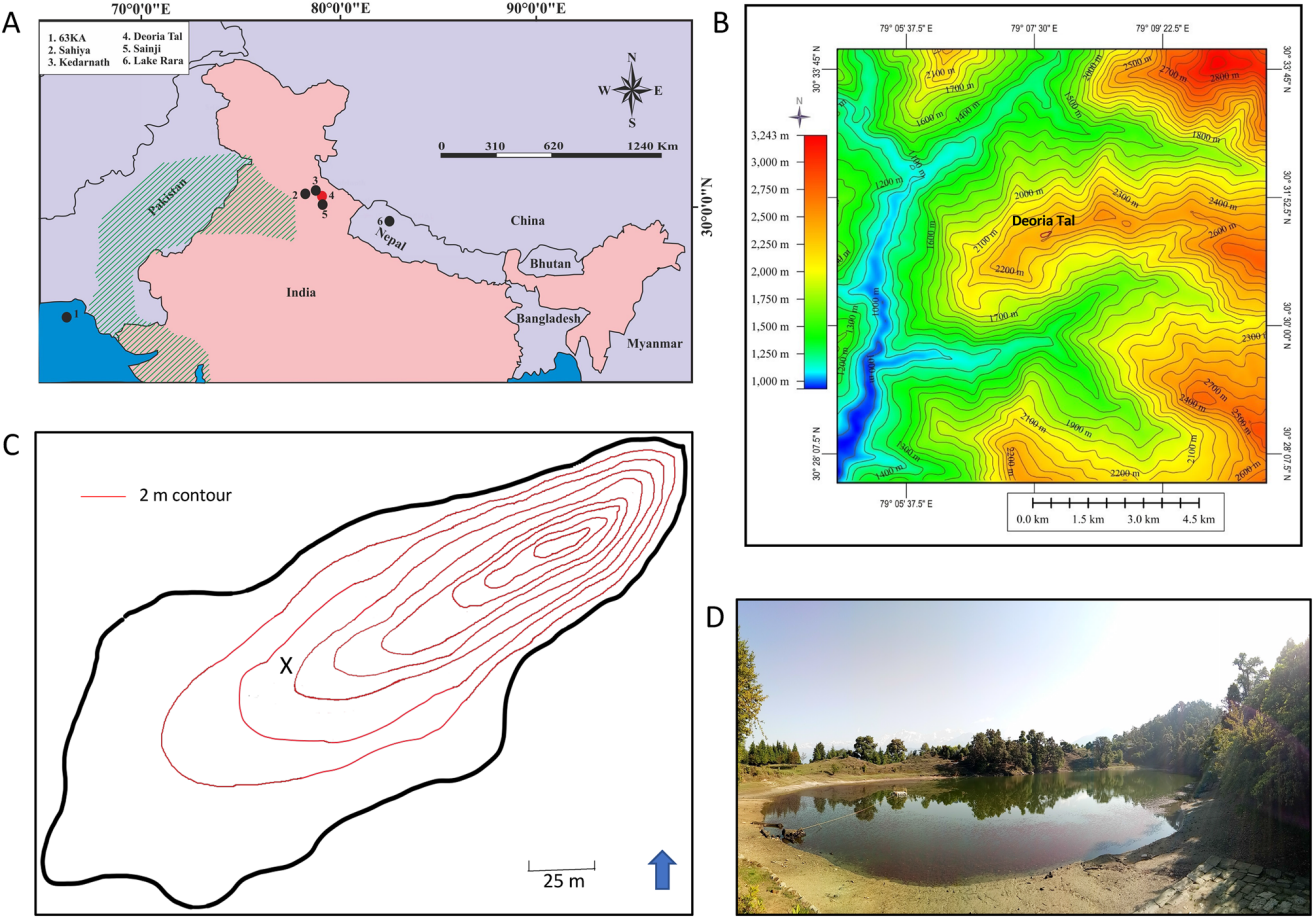
Study area. (**A**) Location of records discussed in text: 63KA^11^; Sahiya Cave^4^; Kedarnath Peat Sequence^12^; Deoria Tal (this study); Sainji Cave^13^; Lake Rara^14^ and the spatial extent of the Indus Civilization (green hatching). (**B**) Topographic context for Deoria Tal (outlined in red). (**C**) Estimated bathymetry of the lake (determined by weighted sounder and based on sketch map created in the field) and the coring location (X). (**D**) Photo of Deoria Tal illustrating the location of *Trapa* in the littoral of the lake, the coring platform, and the snow-covered peaks of the Himalaya in the background. (**A**) and (**B**) were created using Corel Draw Graphics Suite (version × 8; www.corel.com). (**C**) was created using Adobe Photoshop CC (version 20; https://www.adobe.com/products/photoshop.html).

At 4200 cal yr BP, northern India was occupied by the well-established Indus civilization^9,10^. The Indus cvilization, also referenced as the Indus civilization, was a socially complex, agrarian and highly urbanized civilization located along the Indus and Ghaggar-Hakra river valleys (Fig. 1). During the Late Mature phase^15^ (between ~ 4200 and ~ 3900 cal yr BP), the Indus civilization was characterized by well-developed infrastructure including large cities with extensive sanitation facilities^16^ and agricultural and water delivery systems heavily reliant on hydraulic engineering^17^. A distinguishing Late Mature phase characteristic is that it spans the onset of the Indus civilization’s “collapse” or “metamorphosis”, which involved demographic and spatial contraction comprised of de-urbanization coinciding with an eastward migration^15,18–20^. Scholars have proposed a number of causes to account for the decline and eventual disappearance of the Indus civilization, including hydroclimate anomalies, disease, warfare, tectonic-induced changes in the course of major river systems, socio-political instabilities, changes in crop patterns, and/or the disruption of trade networks^21^. Although the specific cause remains unknown, archaeological and paleoclimate studies have identified abrupt climate change and hydroclimate variability, specifically an increase in climate-induced drought episodes from ~ 4200 cal yr BP onwards, as a trigger for the decline of this highly complex civilization^11,18,22–24^.

The ISM is the main source of precipitation for Peninsular India, which includes the region formerly occupied by the Indus civilization. Variations in the strength and spatial extent of the ISM on decadal timescales would have greatly influenced this agrarian society^23^. Previous paleoenvironmental and paleoclimate studies assessing potential links between the Indus civilization’s re-organization and climate include lake sediment-based reconstructions from northern India^25–31^, marine sediment records from the Arabian Sea and Bay of Bengal^11,18,20,32,33^, and stalagmites^4,13,34^ from the region. Other approaches include relating climatologically driven fluvial dynamics to settlement locations^19,35,36^ and inferring climate based on agricultural requirements^17^. Modeling and paleoenvironmental studies suggest that the rapid, step-wise, and sustained decline in population that characterized the Indus civilization, beginning at ~ 4200 cal yr BP, can be attributed to increasing water scarcity associated with a weakening of the ISM and changes in regional hydroclimate^23,24,29^. However, existing records provide inconsistent and sometimes-conflicting evidence of the timing and nature of climate and environmental changes during the mid- to late Holocene, limiting our ability to explicitly link hydroclimatic variability with the archeological evidence of civilization and cultural change in the region^4^.

This study was undertaken to determine if the decline in the Indus civilization occurred during an interval characterized by a sustained decrease in precipitation and increasing hydroclimate variability. Here, we present a sub-decadal-scale reconstruction of hydroclimate variability between 5200 and 200 cal yr BP. We focus on the interval between 5200 and 3000 cal yr BP, which spans the time when the Indus civilization flourished and declined. We utilize non-destructive, radiological approaches, e.g. X-ray fluorescence (XRF) and Computerized Tomography (CT) scans of a well-dated lake sediment core recovered from Deoria Tal, a small, closed basin lake located in the Garhwal Himalaya to: (1) characterize regional hydroclimate variability during the mid- to late Holocene; (2) determine if evidence of an abrupt, short-lived climate event at 4200 cal yr BP is present at this site; and (3) assess the correspondence between hydroclimate variability and Indus civilization re-organization during the late Holocene.

## Study site

Deoria Tal (30.5222° N, 79.1277° E) is located on a hilltop (2393 m a.s.l.) in the northern Indian state of Uttarakhand (Fig. 1). The lake is situated in a high-grade metamorphic terrain above the Main Central Thrust (MCT) zone in the Garhwal Himalaya and is underlain by three major lithotectonic groups^37,38^. The lithotectonic groups are delineated by three main thrusts—Bhulkund Thrust, Okhimath Thrust, and Banswara Thrust (from north to south). Deoria Tal is located between the Bhulkund Thrust and the Okhimath Thrust and is underlain by porphyritic gneiss with mica schist and granite also present^39^ (Fig. S1). Like quite a few other lakes in the Indian Himalaya^40,41^, Deoria Tal appears to have formed as a result of neo-tectonic activity.

The majority of the Indian subcontinent, which is characterized by warm, wet summers, is largely influenced by the ISM, with approximately 70% of the total annual precipitation falling between June and September^42^. The seasonal migration of the Intertropical Convergence Zone (ITCZ) and its movement southward during the late boreal summer limits the amount of precipitation that falls over most of India during the winter and spring, with the northeast coast and northwestern India being the exception^43^. Lying south of the ITCZ, the major source of precipitation for Deoria Tal is the ISM with average annual rainfall totaling around 200 cm^44^ and daily amounts of precipitation ranging between 8 and 10 mm/day^45^. Deoria Tal also receives lesser amounts of precipitation from extra-tropical cyclones, also known as Western Disturbances, between December and February^46^. Western Disturbances, which are frontal systems that originate over the North Atlantic, Mediterranean, and Black Sea regions during the boreal winter and spring, contribute a significant amount to total annual precipitation in the far north and northwest of India^47^. On inter-annual to inter-decadal timescales, variations in the North Atlantic Oscillation (NAO), the Atlantic Multidecadal Oscillation (AMO), the El Nino–Southern Oscillation (ENSO), and the Indian Ocean Dipole (IOD), can also modulate ISM behavior and strength^23,30,48–51^.

Deoria Tal is a small (surface area = 2.7 ha), closed basin lake, surrounded by a relatively small catchment (11 ha). Limnological data for the lake are available in the Supplemental Material (Table S1). Deoria Tal is moderately deep (depth = 16 m) (Fig. 1) and was strongly stratified when sampled on May 18, 2018. Surface water temperature was 19.1 °C, with a bottom water temperature of 6.4 °C (Table S1). A mat of water chestnut (genus: *Trapa*), which requires relatively shallow (1–4 m), nutrient rich water^52^, covers the littoral of Deoria Tal. Human impact on the lake system is currently relatively limited as it is protected by the Uttarakhand Forestry Department; however, human influence has been greater in the past. The expansion of the Indus civilization eastward through the Ganga Plain began at ~ 5000 cal yr BP^53^ and Demske et al.^31^ suggest, based on their pollen analysis of a lake sediment core from a nearby site, Badanital (located 20 km east of Deoria Tal), that migrants may have potentially arrived in the region at ~ 4000 cal yr BP. Other evidence of human influence includes the presence of the well-documented Painted Grey Ware (PGW) culture and large-scale cultivation between 2000 and ~ 1300 cal yr BP in the region^31^.

## Results

### Chronology and sedimentation rates

**Figure 2:**
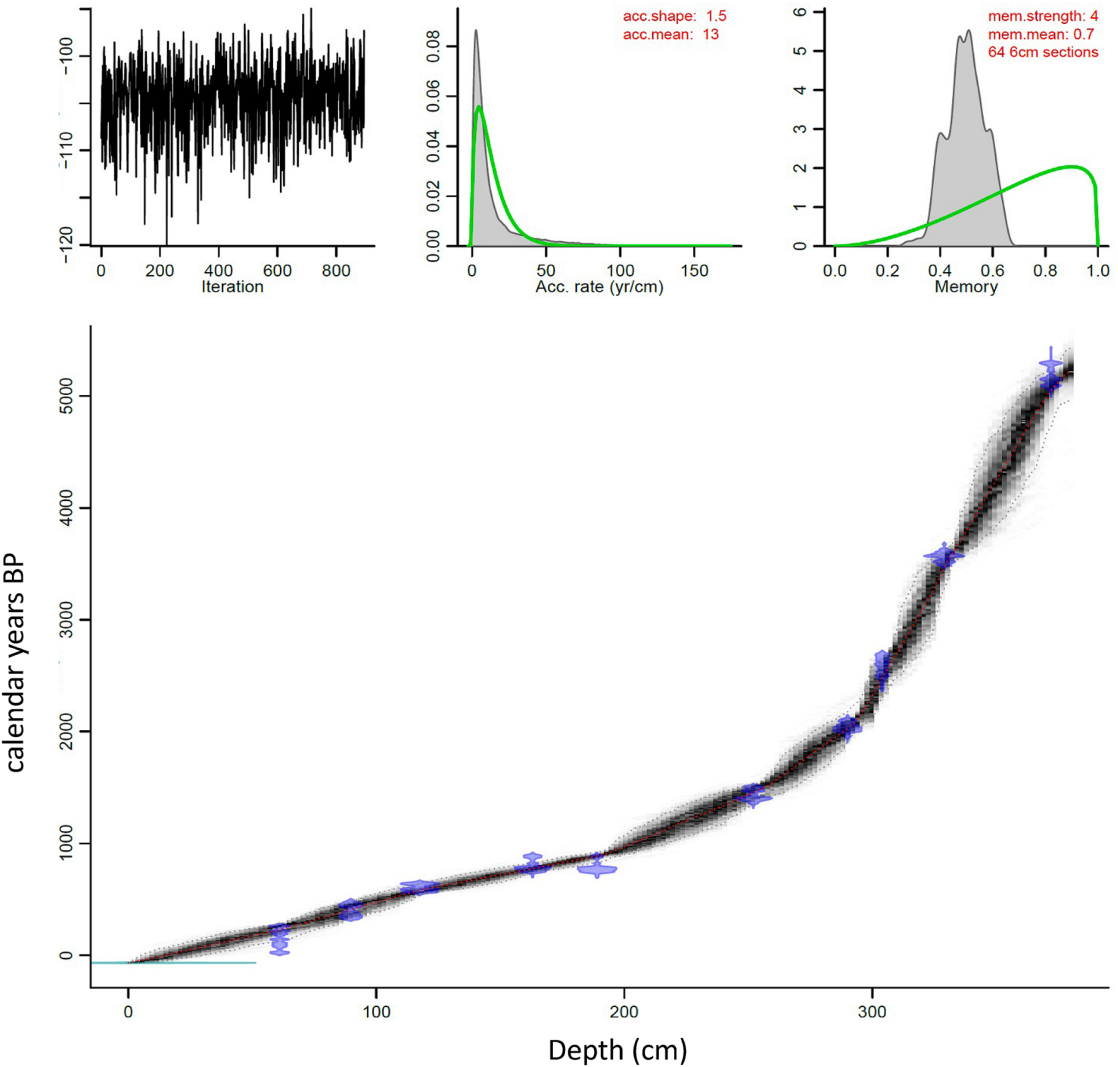
Age-depth model output from BACON^54^. The depth of the lake sediment core (x-axis) is plotted from 0 to 380 cm and the calibrated ages (y-axis) are plotted in cal yr BP. Each of the water chestnut seed cases (blue) are plotted against the age-depth curve (black) with the dotted lines marking the 2σ range of uncertainty.

The age-depth model, developed in BACON^54^, indicates that the base of the sediment core extends back to ~ 5200 cal yr BP (Table 1; Fig. 2). An extant *Trapa* seed case returned a modern radiocarbon date with a percent Modern Carbon (pMC) equal to 101.9. This result indicates that the ^14^C dates obtained on the *Trapa* seed cases do not need to be corrected for a reservoir effect. A prominent increase in the rate of sedimentation occurs at ~ 1500 cal yr BP. The rate of sedimentation goes from ~ 17 years/cm at 1600 cal yr BP (264 cm) to 10 years/cm at 1500 cal yr BP (256 cm). The timing of this change in sedimentation, which aligns with the onset of zone DRL-3 (1500 cal yr BP; 256 cm) and a decrease in the flux of detrital elements, e.g. Rb, Sr,Ti and Zr, corresponds to the occurrence of large-scale cultivation in the region^31^.

**Table 1:** Radiocarbon dates from Deoria Tal. All samples calibrated using OxCal 4.3^55^ with the ages reported using the 2σ range derived from Reimer et al.^56^.

Sample location	Depth (cm)	Sample material	CAIS ID#	pMC	± PMC error	δ^13^C	^14^C yr. BP	^14^C ± error	Cal yr. AD/BC (2σ)	Curve
Modern	0	Seed case	36821	101.99	0.25	− 26.81	Modern	-	1955.48–1957.44 AD	Bomb13 NH3
PT	61	Seed case	37904	98.26	0.23	− 25.41	140	20	1670.94–1707.92 AD 1719.06–1779.3 AD 1799.3–1826.72 AD 1832.54–1888.08 AD 1913.8–1942.9 AD 1952.56–1953.86 AD	Bomb13 NH3
PT	89.67	Seed case	37905	95.76	0.23	− 23.24	350	20	1462–1529 AD 1545–1635 AD	IntCal13
LC1A	117.5	Seed case	37906	92.58	0.23	− 25.11	620	20	1294–1330 AD 1339–1397 AD	IntCal13
LC1A	163	Seed case	37907	89.6	0.22	− 26.57	880	20	1049–1084 AD 1124–1137 AD 1150–1218 AD	IntCal13
LC1B	189	Seed case	37908	89.79	0.22	− 24.85	870	20	1052–1080 AD 1151–1220 AD	IntCal13
LC1B	252	Seed case	37909	82.66	0.2	− 24.24	1530	20	429–494 AD 511–517 AD 529–595 AD	IntCal13
LC1C	290	Seed case	37910	77.31	0.19	− 26.03	2070	20	166–41 BC	IntCal13
LC1C	304	Seed case	40239	73.53	0.19	− 26.22	2470	20	764–508 BC 500–491 BC	IntCal13
LC1C	329	Seed case	40238	66.1	0.18	− 26.55	3330	20	1683–1672 BC 1666–1597 BC 1588–1532 BC	IntCal13
LC1D	372	Seed case	37104	56.8	0.16	− 24.99	4540	25	3365–3311 BC 3295–3287 BC 3275–3265 BC 3239–3105 BC	IntCal13

### Chemostratigraphy

**Figure 3:**
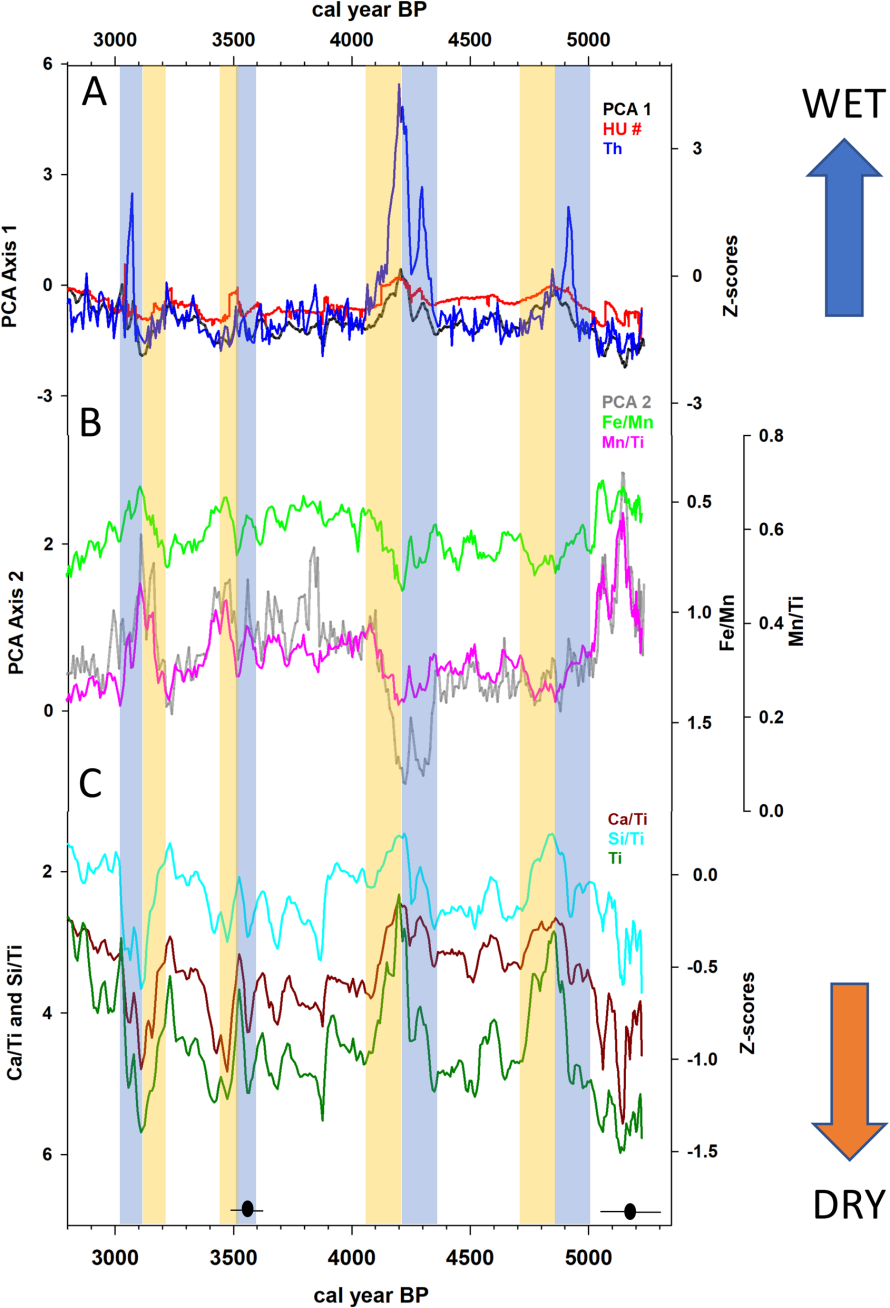
(**A**) Principal component analysis (PCA) Axis 1 (black), HU number (red), and Th (z-scores) (blue); (**B**) PCA Axis 2 (grey), Fe/Mn (reversed scale) (green) , and Mn/Ti (pink); (**C**) Ca/Ti (reversed scale) (red ochre), Si/Ti (reversed scale) (baby blue), and Ti (z-scores) (dark green). The vertical tan and blue-shaded boxes represent intervals characterized by negative and positive hydroclimate anomalies, respectively. The mean value (black circle) and 2-sigma range (black line) for the calibrated ages, based on the radiocarbon dates available for this portion of the core, are also depicted.

The results of a Principal Components Analysis (PCA) of the pXRF-derived elemental data was used to divide the core into four distinct zones: DRL-1 (5200–3000 cal yr BP) is characterized by fluctuating and anti-phased PCA axes 1 and 2 scores; DRL-2 (3000–1500 cal yr BP) is characterized by decreasing PCA Axis 1 and increasing PCA Axis 2 scores; DRL-3 (1500–800 cal yr BP) is characterized by decreasing PCA axes scores; and DRL-4 (800 cal yr BP–present) is characterized by anti-phased PCA axes scores (Fig. S2). The pXRF provides returns for ten elements: silicon Kα, potassium Kα, calcium Kα, titanium Kα, manganese Kα, iron Kα, rubidium Kα, strontium Kα, zirconium Kα, and thorium Lα (from hereon, referenced by the respective elemental symbol) (Fig. S3). The Spearman’s correlation coefficients indicate the existence of a statistically significant correlation between all of the elements (Fig. S4). A PCA with the 10 measured elements and five ratios (Fe/Mn, Zr/Rb, Si/Ti, Ca/Ti and Mn/Ti) indicates that approximately 78% of the variance in the dataset could be explained by the first two principal component axes (Fig. S5). PCA Axis 1, which captures 61% of the variance in the elemental dataset, is strongly correlated with K, Ti, Fe, Rb, Sr, Zr, and Th (Table S2). PCA Axis 2, which captures an additional 16% of the variance, is strongly correlated with Mn, Fe/Mn and Mn/Ti. A plot of the PCA axes against time indicates notable deviations in the elemental data centered at 4850, 4200, and 3100 cal yr BP (Fig. 3).

DRL-1 (380–318 cm; 5200–3000 cal yr BP): Elemental returns in DRL-1 are largely driven by variations in the elements strongly correlated with PCA Axis 1 (K, Ti, Fe, Rb, Sr, Zr, and Th) while smaller magnitude changes are evident in Si, Ca, and Mn through the early portion of this zone (Figs. S2, S3). Prominent fluctuations in the elemental counts are centered at 4850, 4200, and 3100 cal yr BP with a less pronounced event evident at 3500 cal yr BP. The events centered at 4850, 4200 and 3500 cal yr BP are initially characterized by increasing PCA Axis 1 scores, HU number (measure of radiodensity calibrated according to the Hounsfield Scale), Th, and Fe/Mn and decreasing PCA Axis 2 scores, Mn/Ti, Ca/Ti, and Si/Ti between 5000 and 4850, 4350 and 4200 and 3600 and 3500 cal yr BP, respectively (Figs. 3, S6, S7). Each of these events is then followed by an interval characterized by decreasing PCA Axis 1 scores, HU number, Th, and Fe/Mn and increasing PCA Axis 2 scores, Mn/Ti, Ca/Ti, and Si/Ti from 4850 to 4700, 4200–4050 and 3500 to 3400 cal yr BP, respectively. The last event, between ~ 3200 and 3000 cal yr BP, is initially defined by decreasing PCA Axis 1 scores, HU number, Ti, and Fe/Mn and increasing PCA Axis 2 scores, Mn/Ti, Ca/Ti, and Si/Ti between ~ 3200 and 3100 cal yr BP; followed by increasing PCA Axis 1 scores, HU number, Th, and Fe/Mn and decreasing Mn/Ti, Ca/Ti, and Si/Ti between 3100 and 3000 cal yr BP.

DRL-2 (318–256 cm; 3000–1500 cal yr BP): Prominent fluctuations in the elemental counts are centered at 2000 and 1650 cal yr BP (Figs. S2, S3). The events between 2100 and 1900 cal yr BP and 1700 and 1600 cal yr BP are initially characterized by decreasing PCA Axis 1 scores, HU number, Th, and Fe/Mn with increasing PCA Axis 2 scores, Mn/Ti, Ca/Ti, and Si/Ti (Figs. S6, S7). These events are followed by an interval characterized by increasing PCA Axis 1 scores, HU number, Th, and Fe/Mn and decreasing PCA Axis 2 scores, Mn/Ti, Ca/Ti, and Si/Ti between 2000 and 1900 cal yr BP and 1650 and 1600 cal yr BP, respectively.

DRL-3 (256–169 cm; 1500–800 cal yr BP): The PCA results are more variable in DRL-3 relative to DRL-1 and DRL-2 with large magnitude shifts in the elemental returns centered on 1450 and 1250 cal yr BP (Figs. S2, S3). The event between 1500 and 1400 cal yr BP is initially characterized by lower PCA Axis 1 scores, HU number, Th, and Fe/Mn and increasing PCA Axis 2 scores, Mn/Ti, Ca/Ti, and Si/Ti between 1500 and 1450 cal yr BP; followed by increasing PCA Axis 1 scores, HU number, Th, and Fe/Mn and decreasing PCA Axis 2 scores, Mn/Ti, Ca/Ti, and Si/Ti between 1450 and 1400 cal yr BP (Figs. S6, S7). The event between 1350 and 1150 cal yr BP is initially characterized by lower PCA Axis 1 scores, HU number, Th, and Fe/Mn and increasing Ca/Ti and Si/Ti between 1350 and 1250 cal yr BP; followed by increasing PCA Axis 1 scores, HU number, Th, and Fe/Mn and decreasing Ca/Ti and Si/Ti between 1250 and 1150 cal yr BP.

DRL-4 (169–50 cm; 800–180 cal yr BP): This zone is characterized by the greatest amount of variance in the PCA and CT scan results. The PCA identifies multiple shifts in the elemental returns, the most prominent of which are centered at 500, 450, and 400 cal yr BP (Figs. S2, S3). These events are characterized by lower PCA Axis 1 scores, HU number, and Fe/Mn and elevated PCA Axis 2 scores, Mn/Ti, Ca/Ti, and Si/Ti. The interval between 400 and 180 cal yr BP is also characterized by highly variable elemental counts and sediment density, as inferred from HU number (Figs. S6, S7).

## Discussion

Given our interest in assessing the relationship between hydroclimate variability and documented changes in the Indus civilization we focus our efforts on developing a holistic paleoclimate reconstruction spanning the interval captured by DRL-1 (5200–3000 cal yr BP), paying particular attention to the hydroclimate events evidenced at 4200 and 3100 cal yr BP.

### Environmental interpretation of elemental and radiological proxies

Hydroclimatic regimes can influence the allogenic processes responsible for shaping the detrital flux to a lake. Typically, in subtropical environments during a wetter precipitation regime an elevated flux of coarse-grained sediment will be deposited within the lake^57–59^. As a means to distinguish anomalous climatic conditions in lacustrine records, general associations have been recognized between specific elements, as identified by XRF, and grain size at numerous sites in varying geologic and hydroclimatic conditions^60^. Existing grain size-element associations enable the development of specific elemental ratios that further aid environmental interpretation of hydroclimate variability. Fluctuations in Ti, which are associated with grain-size variations, have been used to reliably reconstruct rainfall and run-off^61,62^ and detrital input^63,64^ in varied settings. At Deoria Tal, PCA Axis 1 is inferred to primarily reflect variations in detrital influx. The elements most strongly correlated with PCA Axis 1 (K, Ti, Fe, Rb, Sr, Zr, and Th) have been identified has common constituents of detrital-sourced sediment^14,58,65–69^. The grouping of Ti with PCA Axis 1 and the strong Spearman’s correlation coefficients between Ti and Rb (ρ^2^ = 0.94), and Zr (ρ^2^ = 0.92), further supports the inference that variations in Ti are related to detrital input from the catchment^70^ (Figs. S4 and S5). Changes in sediment density, as captured by the HU number, are used to provide additional support for the PCA-based inferences of detrital flux^71,72^. A higher HU number is reflective of increasing sediment density and is inferred to occur during intervals characterized by elevated clastic/minerogenic input (i.e. increasing detrital flux). Decreasing HU numbers are inferred to reflecting lower sediment density and reduced detrital input.

PCA Axis 2 is inferred to reflect variations in hypolimnetic oxygen conditions. Hypolimnetic oxygen conditions are often reconstructed using iron^28^ and manganese^54,62^. The relative abundance of iron and manganese is influenced by redox potential in the benthic zone of aquatic systems^53,54,57,58,64^, which in turn is influenced by hypolimnetic oxygen conditions. The element most strongly correlated with PCA Axis 2 is Mn, which is strongly influenced by redox conditions^66,73^ (Fig. S5). During periods of stratification, ventilation of the bottom water is limited resulting in the reduction of hypolimnetic oxygen concentration. Higher lake levels reduce the ability of the wind to mix (ventilate) the entire water column of the lake and facilitates the onset of anaerobic conditions in the hypolimnion; whereas, lower lake levels would facilitate wind-mixing of the water column and increase oxygen supply to the bottom water. Fe/Mn and Mn/Ti ratios are commonly utilized as proxies for hypolimnetic oxygen conditions and redox conditions^74,75^. It is expected that Fe/Mn would decrease during periods characterized by abundant hypolimnetic oxygen concentration (lower lake level, weakened/no stratification) given that Mn is highly insoluble in oxic conditions and increasingly soluble, relative to Fe, in reducing (anoxic) environments^76^. It is important to note that redox potential in freshwater, is controlled by a suite of variables (e.g. dissolved oxygen, pH, temperature, organic matter, and nutrient availability), all of which can be influenced by changing limnological conditions including variations in lake productivity and lake level^77^.

In variable environments, such as lake systems, it is standard to normalize the variations of the pXRF-derived element counts for Ca and Si using Ti, an element that is not affected by biological or redox processes^78^. Ca/Ti and Si/Ti are used to decipher lake level and changes in autochthonous productivity, respectively (Fig. 3). Calcium in lake sediments may be related to both carbonate weathering in the catchment and in-lake precipitation of carbonate. Carbonate is precipitated when lake waters become saturated with respect to cCaCO_3_, which can occur when solutes become increasingly concentrated in response to a reduction in lake volume^79^. Numerous studies have documented that Ca/Ti can serve as a sensitive hydrological proxy with high Ca/Ti values indicative of lower lake level and evaporative enrichment and low Ca/Ti values reflecting increasing lake volume^80,81^. The PCA analysis indicates that Ca/Ti loads negatively on the axis capturing detrital flux (PCA Axis 1), providing support that the variations in Ca are primarily driven by within lake processes rather catchment processes (Fig. S5). This, together with the existence of a relatively weak correlation between Ca and detrital elements (i.e. Ti, Rb, Zr and Sr) (see Fig. S4) supports the claim that the Ca present in the lake sediment is related to the autochthonous precipitation of carbonate. Treatment of sediment samples with HCl for on-going stable isotope analysis indicates the presence of CaCO_3_ throughout the core. Si/Ti can be used as a proxy for biogenic silica with elevated Si values representing an increase in diatom productivity^64,82,83^ or as an indicator of clastic input^75,84^. The PCA analysis indicates that Si/Ti loads negatively on PCA Axis 1 (Fig. S5), suggesting that the variations in Si evidenced in the sediment are driven by autochthonous biogenic productivity rather than clastic input. We infer that elevated diatom productivity at Deoria Tal, as evidenced by high Si/Ti, occurred during intervals characterized by weakened stratification (due to the increasing effectiveness of wind mixing of a shallower lake), which increased nutrient availability in the surface waters. The anti-phased relationship between Si/Ti and Fe/Mn between 5000 and 3000 cal yr BP (Fig. 3) supports this inference with increased biogenic productivity (reflected by elevated Si/Ti) occurring during intervals characterized by lower lake levels, weakened stratification and abundant hypolimnetic oxygen concentration (reflected by reduced Fe/Mn)^85^.

### Paleoclimate reconstruction

The event between 4350 and 4050 cal yr BP is initially characterized by elevated precipitation and a positive hydroclimate anomaly (Fig. 3). This event is larger in magnitude than the event centered at 4850 cal yr BP. This inference is supported by the elevated Fe/Mn ratio suggesting the occurrence of anoxic conditions in the bottom water between 4350 and 4200 cal yr BP. The existence of anoxia in the deep water, also supported by lower Mn/Ti, is likely a response to a deepening of the lake and strengthened thermal stratification, both of which would reduce water column mixing and thereby reduce oxygenation of the bottom water. As with the 4850 cal yr BP event, reduced diatom productivity, reflecting limited nutrient availability, is also evident. The increase in allogenic detrital input between 4350 and 4200 cal yr BP, inferred from the elevated counts for PCA Axis 1, Th and HU number provide further support for this inference, as increased precipitation would increase erosion in the catchment and the transport of detrital material to the lake.

**Figure 4:**
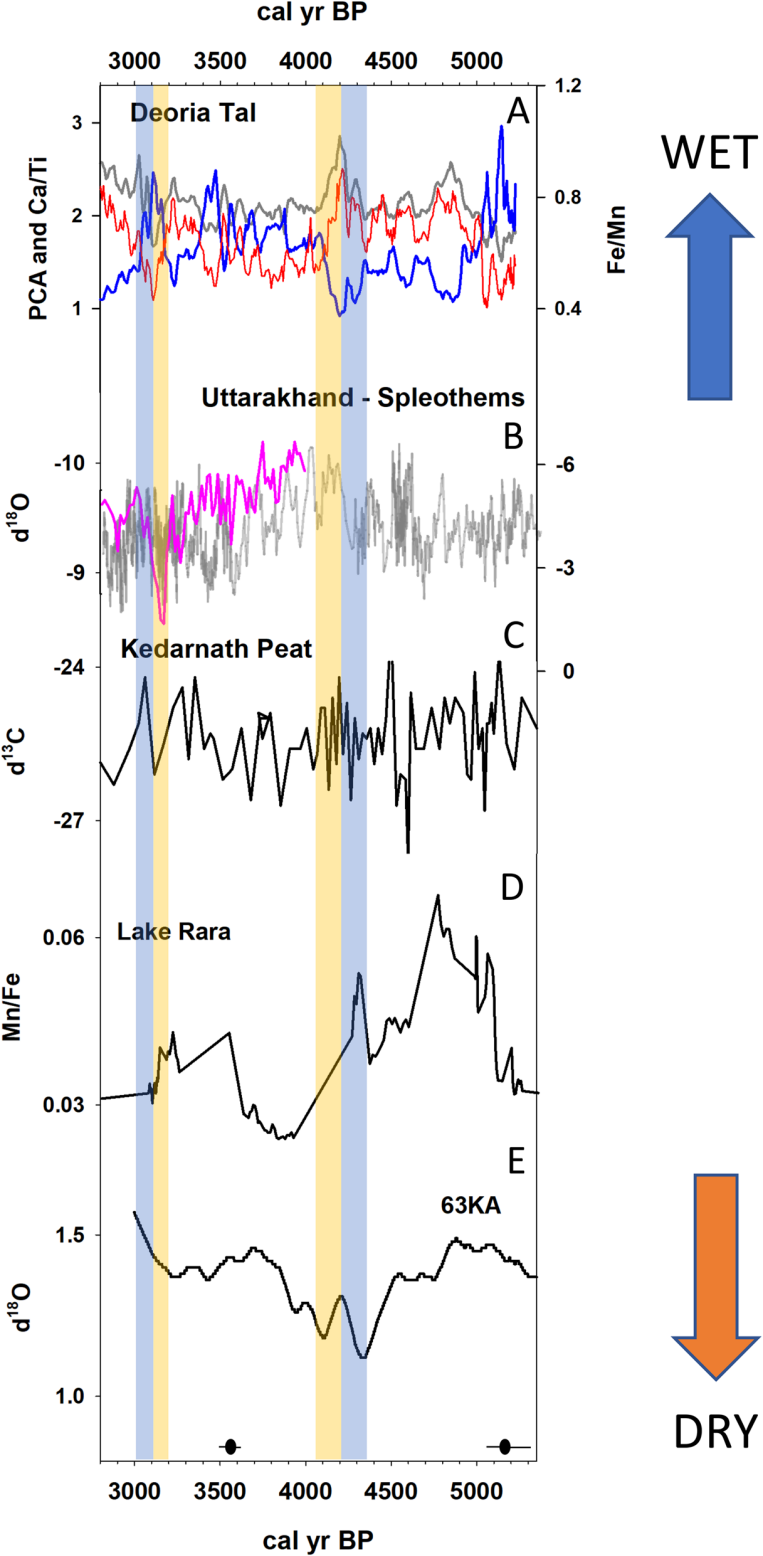
Synthesis figure depicting paleoclimate records from the region. (**A**) PCA Axis 1 scores (grey), Ca/Ti (blue) and Fe/Mn (red), this study; (**B**) δ^18^O of speleothems (reversed scale) from Sainji Cave, Uttarakhand^13^ (pink, right-hand scale); Sahiya Cave^4^ (cyan, left-hand scale); (**C**) δ^13^C from Kedarnath peat bog, Uttarakhand^12^; (**D**) Mn/Fe from Lake Rara, Nepal^14^; (**E**) δ^18^O (*Neogloboquadrina dutertrei*_315-400um_ − *Globigerinoides ruber*_400-500um_) from marine core 63KA, Arabian Sea^11^. The vertical tan and blue-shaded boxes represent intervals characterized by negative and positive hydroclimate anomalies, respectively. The mean value (black circle) and 2-sigma range (black line) for the calibrated ages, based on the radiocarbon dates, available for this portion of the core are also depicted.

Evidence of wet conditions in northern India is documented at Sahiya Cave, with variations in δ^18^O inferred to reflect an interval of elevated ISM between at 4300 and 4150 cal yr BP^4^ (Fig. 4). An abrupt increase in wind mixing and oxygenation of lake water between 4400 and 4200 cal yr BP at Lake Rara, in western Nepal, is associated with a strengthened ISM during this interval^14^ (Fig. 4). Marine records from the Arabian Sea suggest that monsoonal precipitation peaked between 4500 and 4300 cal yr BP^18^, with evidence that the increase in precipitation during this interval was driven primarily by an increase in the IWM^11^ (Fig. 4). At 4200 cal yr BP the parameters outlined above shift in the opposite direction at DRL suggesting the onset of drier conditions, leading to a negative lake mass balance and a lowering of lake level. An abrupt decrease in effective moisture between 4200 and 4000 cal yr BP is documented in multiple records from South Asia. Peak drying is evident at 4100 cal yr BP at paleolake Kotla Dahar, in northwest India^24^; between 4200 and 4000 cal yr BP at Lonar Lake, in central India^25^; and at 4200 cal yr BP at Tso Kar Lake, in northern India^28^. A shift from pluvial conditions to a variable ISM characterized by multiple multi-decadal scale droughts is inferred to have occurred between 4200 and 4050 cal yr BP in northern India^86^. The onset of dry conditions at this time is also documented at Lake Rara in western Nepal^14^ and at Mawmluh Cave in northeast India^34^. Marine records indicate a brief interval of increasing surface water salinity in the Bay of Bengal at 4200 cal yr BP^32^; the occurrence of a centennial-scale reduction in the discharge of the Indus River at ~ 4200 cal yr BP^18^; and a surface water salinity episode in the northern Arabian Sea, inferred to reflect a concurrent reduction in the IWM and ISM beginning at 4100 cal yr BP^11^.

The event between 3200 and 3000 cal yr BP is inferred to initially reflect the occurrence of a severe drought episode followed by a notable increase in effective moisture at Deoria Tal. This inference is supported by the existence of low Fe/Mn and high Mn/Ti between 3200 and 3100 cal yr BP, which suggest that the bottom waters were well oxygenated, likely resulting from the increased effectiveness of wind-driven water column mixing in a shallow lake. The negative excursion in Ti provides support for lake drawdown during this interval as well, as lower counts of Ti have been associated with drought conditions in subtropical environments^57^. The elevated Ca/Ti values, which peak at 3100 cal yr BP, provide additional support for the inference of lower lake levels at this time. An increase in Ca in lake sediment is expected to occur when lake water becomes saturated with respect to calcium, which can occur with reduced lake volume^79^. The increase in diatom productivity, resulting from an increase in nutrient availability due to wind mixing of the shallow lake, provides further support for the inferred reduction of lake levels between 3200 and 3100 cal yr BP. Together with the PCA Axis 1 data, these lines of evidence indicate that this event was characterized by a hydroclimate anomaly that was large enough to significantly lower lake levels. The presence of visibly coarser materials along with an abrupt increase in the PCA Axis 1 scores and HU number at 3100 cal yr BP, suggest that this drought episode was terminated abruptly at 3100 cal yr BP and was followed by a positive hydroclimate anomaly and increasing lake levels.

Evidence of a transition to increasingly arid conditions at ~ 3200 cal yr BP is evident in other regional records with a decadal-scale interval of lowered effective moisture^29^ leading to forest thinning^44^ and marked changes in vegetation due to fire and human activity^31^. This drought episode and the subsequent increase in aridity is very well expressed in a speleothem record from nearby Sainji Cave^13^ (Fig. 4) and a peat sequence from Kedarnath^12^ (Fig. 4). Nakamura et al.^14^ identify a brief decline in the ISM occurring at 3100 cal yr BP (Fig. 4), signaling a transition to a drier regime characterized by a weakened monsoon driven by a reduction in solar insolation^87^. In coastal areas, the dry pulse at 3200 cal yr BP favored the expansion of salt tolerant mangroves^88^. At approximately 3000 cal yr BP this arid interval was terminated^17,44,89^. Following this transition, our record supports the inference of a trend towards wetter conditions, as evidenced in other regional records^89,90^, between 3000 and ~ 2500 cal yr BP. Importantly, the episode of late Holocene peak aridity captured by the Sainji Cave speleothem at ~ 3150 cal yr BP and our record at Deoria Tal coincides with the timing of societal collapse and the disappearance of Indus culture in northwest India^15^.

### Hydroclimate variability and Indus civilization re-organization at 4200 cal yr BP

Broadly, DRL-1 (5200–3000 cal yr BP) lies within the middle of a climatic regime characterized by a weakening ISM^4,68^. The trend of decreasing precipitation though the interval captured by DRL-1 was not gradual nor unidirectional. Notable hydroclimate anomalies characterized this interval with distinctive, discrete multi-decadal to centennial-scale episodes of fluctuating hydroclimate evident at Deoria Tal. The interval between 5000 and 3000 cal yr BP is of particular interest because it spans the interval that captures the rise, expansion, and eventual contraction of the Indus civilization^8^. The Indus civilization, which reached its apogee between 4300 and 3900 cal yr BP, consisted of several thousand communities along the Indus River and the Ghaggar-Hakra River by 4200 cal yr BP^91,92^. The Indus civilization’s agriculture system was dependent on flooding rather than precipitation directly^19^. The rise of the cultural complex associated with the Indus civilization, between 5200 and 4600 cal yr BP, occurs during an interval characterized by a trend of decreasing intensity of ISM and IWM precipitation. This “Goldilocks” scenario, which lasted from approximately 5200 to 4200 cal yr BP, allowed for crops to be planted in areas inundated frequently enough by floods of sufficient magnitude to provide needed irrigation but not so frequently or by such large magnitude floods that crops were damaged^20^.

According to Staubwasser et al.^18^, the 4200 cal yr BP event impacted the agricultural base of this highly centralized agrarian society, which likely contributed to its geographic and demographic contraction and may have ultimately led to demise of their civilization. Modeling and paleoenvironmental studies suggest that the rapid, step-wise, and sustained decline in population that characterized the Indus civilization, which began at ~ 4200 cal yr BP, can be attributed to increasing water scarcity associated with changes in regional hydroclimate and a weakening of the ISM^13,18,23,24,29^. This hypothesis is supported by meteorological observations (1961–1990), which indicate that ~ 80% of total mean annual precipitation that falls in the ISM dominated region falls between June and August^23^. However, recent research has also highlighted the potential contribution of winter precipitation to the overall precipitation regime in this region during the mid- to late Holocene^11,20^. The interval between 4500 and 4100 cal yr BP was characterized by fluctuations in the strength of the IWM^11,20,28,88^, with evidence of a strengthened IWM between 4500 and 4300 cal yr BP and a weakened IWM from 4300 to 4100 cal yr BP, followed by a step- shift at 4100 cal yr BP. Our record supports the inference of positive hydroclimate anomaly peaking at 4200 cal yr BP in the Garhwal Himalaya and the onset of lake drawdown and a severe centennial-scale drought beginning at 4200 cal yr BP. This study cannot distinguish whether the precipitation anomalies identified at Deoria Tal during this interval were generated by variations in the ISM and/or the IWM; however, it is notable that the positive hydroclimate anomalies evidenced in this record correspond to intervals of strengthened IWM^11^.

## Conclusion

The multi-proxy record generated from Deoria Tal identifies major hydroclimate anomalies in the Garhwal Himalaya at 4850, 4200, and 3100 cal year BP, with the last two events being particularly noteworthy. A decrease in allogenic detrital input and Fe/Mn and an increase in Mn/Ti, Ca/Ti and Si/Ti is inferred to reflect the onset of drier conditions at 4200 cal yr BP. Shifts in Fe/Mn and Mn/Ti; a negative excursion in the detrital flux; and elevated Ca/Ti provides support for the inference of lower lake levels and the onset of a centennial-scale drought episode between 3200 and 3100 cal yr BP. The drought events documented at Deoria Tal correspond to existing records from the northern portion of the Indian subcontinent, providing additional insight into the spatial expression of mid- to late Holocene hydroclimate anomalies in this highly populated region. This study sheds light on the temporal relationship between hydroclimate anomalies and large scale deurbanization, population decrease and the spatial contraction of Indus civilization in northern India between 4500 and 3000 cal yr BP. This record suggests that the onset of de-urbanization and the deterioration of the Indus civilization at ~ 4200 cal yr BP coincided with largest hydroclimate anomaly evidenced in the Garhwal Himalaya during the last five millennia.

## Materials and methods

### Core recovery and sediment sampling

A sediment core was recovered from Deoria Tal in May 2018 using a modified Livingstone corer deployed from a platform anchored at a depth of 5.65 m (see Fig. 1 for coring location). The core consists of five overlapping drives with the total composite core length equal to 380 cm. The overlapping core drives were correlated using visual stratigraphy (Fig. S8), the results from the pXRF analysis and radiological imagery (Fig. S9). The uppermost sediment was recovered using a plastic tube to ensure that the flocculent surface sediment was captured with minimal disturbance while the stiffer lower sediment was recovered using a stainless-steel barrel. The flocculent, watery nature of the uppermost sediment necessitated that this sediment was sub-sampled in the field (at 0.5 cm intervals) at the time of core recovery to prevent mixing (these samples were not used for radiological analyses). Limnological measurements were made and water samples for water chemistry were also collected at this time (Table S1). Stratigraphical notes were made in the field and later at Kumaun University, Nainital, Uttarakhand, where each drive was longitudinally split, photographed, and described.

### Chronology development

Chronological control for the sediment core recovered from Deoria Tal is based on ten AMS radiocarbon (^14^C) dates obtained on *Trapa* seed cases (Table 1). Additionally, a seed case from *Trapa* extant in Deoria Tal (marked as “modern” in Table 1) was also dated to determine if ‘old’ carbon or a reservoir effect was present. The samples used for radiocarbon dating were rinsed in deionized water, bagged, and labeled before being submitted for analysis at the Center for Applied Isotope Studies (CAIS) at the University of Georgia. The radiocarbon dates were converted to calendar years using OxCal 4.3^55^ with the reported 2σ age ranges following Reimer et al.^56^. An age-depth model was developed using BACON^54^ (Bayesian Accumulation Model, version 2.2), an open-source R code package (Fig. 2).

### Laboratory analyses

Preparation of the cores for elemental analysis using X-ray fluorescence (XRF) was completed at the Environmental Change Lab at the University of Georgia. The sediment cores were scanned using a Bruker Tracer 5i mounted portable XRF (pXRF) with a rhodium tube source at CAIS. A scanning resolution of 2 mm/step was utilized for the pXRF analysis. To detect elements with a low atomic number (Z) a 60 s exposure time of 20 keV, 35 amps, and no filter was employed. To detect high Z elements, a 60 s exposure time of 40 keV, 35 amps, and a Cu 100 µm: Ti 25 µm: Al 300 µm filter was employed^93^. The scan counts, based on a single scan, were initially post-processed using Bruker Artax software to determine the specific high and low Z elements captured by the analysis. All scan count data are presented. The heterogenous composition of lake sediment and the nature of the sediment matrix can affect pXRF readings by differentially influencing the resultant emission spectra. The sediment matrix effects were corrected following standard procedures^78^. Elemental data from the pXRF analysis were normalized using the rhodium Kα peak to minimize the noise variation of the Compton peak. To distinguish natural variation from anthropogenic influence the resultant time-series spectra were normalized using the aluminum Kα record^14,94^. No error estimates are available for the pXRF analysis; however, the measured response rates (Kα) of the elements range from 15,878 to 247 counts/second, which are well above the nominal sensitivity of the Bruker Tracer 5i^95^. Principal component analysis (PCA), of the XRF-derived elemental data (non-transformed, centered and standardized) was utilized to identify which of the elements could account for a statistically significant amount of variance along a reduced number of environmental axes^65,96–98^ (Fig. S5; Table S2). The statistical analyses were undertaken in R^99^ and CANOCO version 4.0^100^.

Computerized Tomography (CT) scans, also known as Computerized Coaxial Tomography (CAT) scans^71^, were completed at the University of Georgia Veterinary Teaching Hospital’s Radiology and Imaging Department on a Siemens Sensation 64 CT scanner utilizing Syngo WinNT 5.1 Service pack 3 imaging processing software. The output from CT scans, known as CT numbers, are calibrated according to the Hounsfield Scale. Hounsfield units (HU) are a dimensionless unit universally used to express CT numbers in a standardized form. HU are a measure of radiodensity based on the linear transformation of the measured attenuation coefficients which are calibrated using the arbitrarily-assigned densities of air (0 HU) and pure water (− 1000 HU). This calibration results in a scale ranging from − 1000 HU for air to + 3000 for metal^101^. HU numbers (or CT numbers) are influenced by bulk sediment density, mineralogy, and porosity^71,102^ and therefore CT scans can be used to identify internal structure within sediment and aid in paleoenvironmental interpretation^72,103^. In this study, an increase in HU number is interpreted to reflect an increase in sediment density resulting from higher clastic/minerogenic input (i.e. elevated detrital flux); a decrease in HU number is interpreted to reflect lower sediment density resulting from a reduction in clastic/minerogenic input (i.e. lower detrital flux). Each core drive was imaged axially with two different exposure settings: Soft (40 HU window level; 300 HU window width) and Bone (400 HU window level; 4000 HU window width) (Fig. S9). The tomograms were compiled using a MATLAB™ package^104^. Presentation of the CT scan HU data (z-scores and standard deviation) is limited to the Soft CT settings output (Fig. S6).

## Supplementary Information

Supplementary Information.
