# Supplementary material for: Hydroclimate change in the Garhwal Himalaya, India at 4200 yr BP coincident with the contraction of the Indus civilization
Source: Sci Rep. 2021 Nov 29;11:23082. doi: 10.1038/s41598-021-02496-5 (PMC8630182; doi:10.1038/s41598-021-02496-5)
Supplement: Supplementary file 1 — Supplementary Information. [file 41598_2021_2496_MOESM1_ESM.pdf]

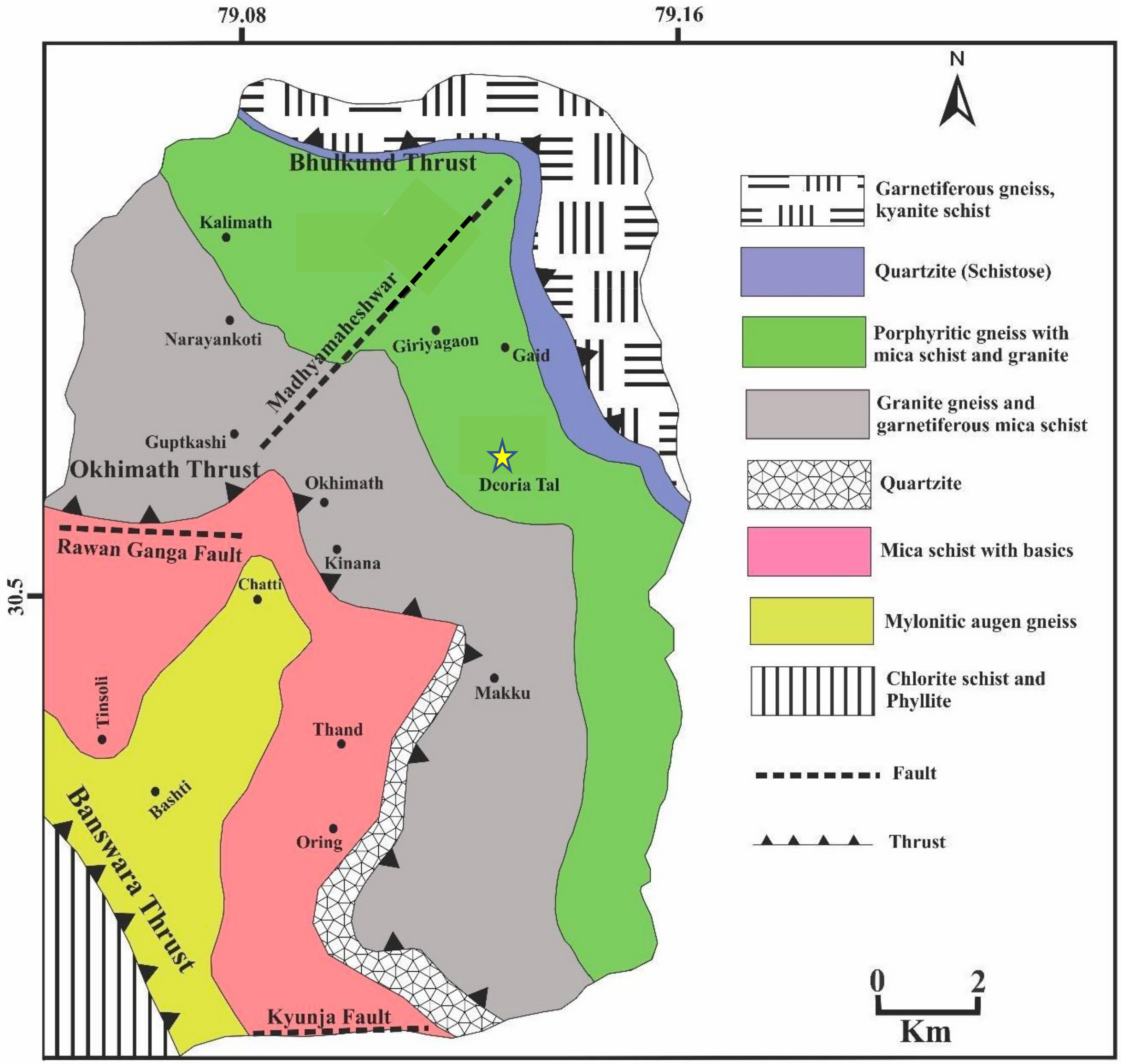

Supplementary Figure 1

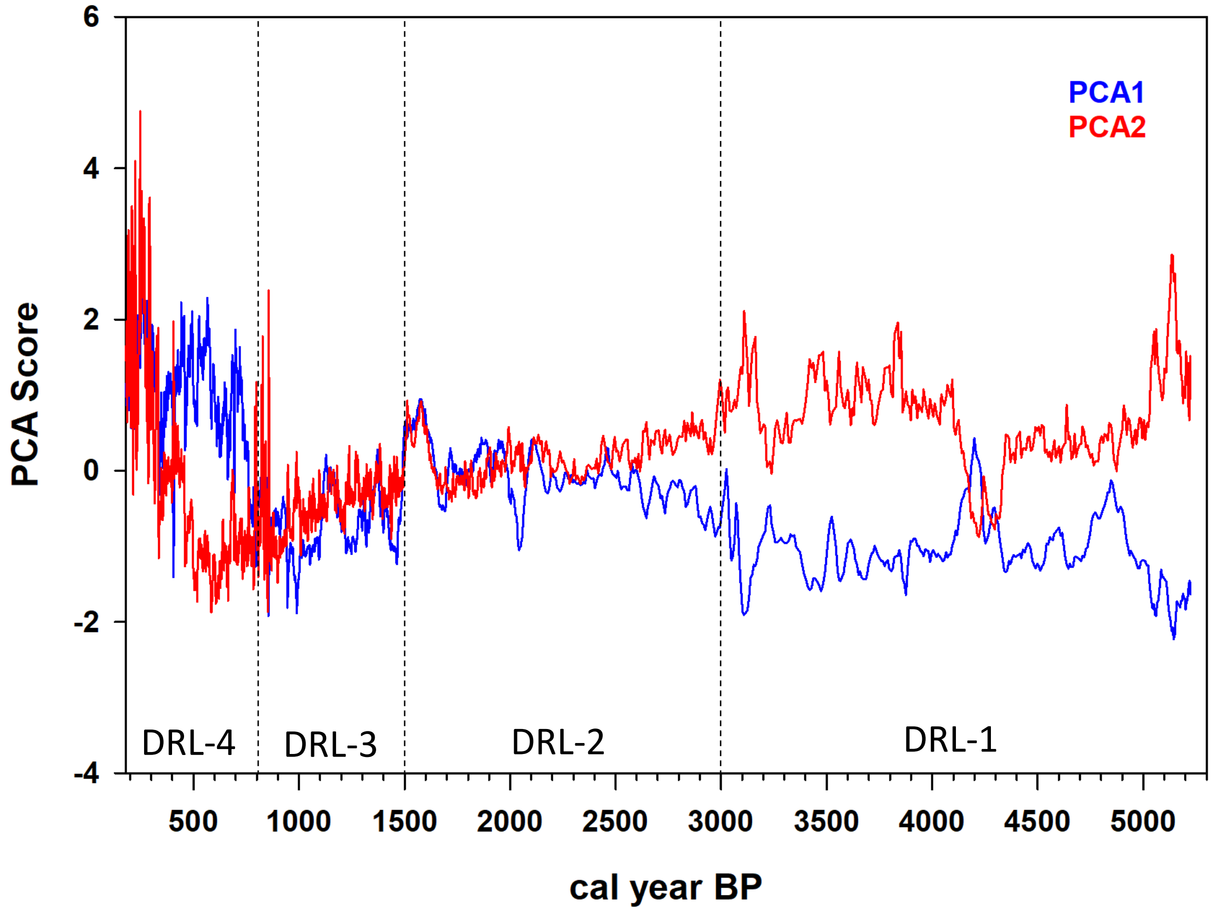

Supplementary Figure 2

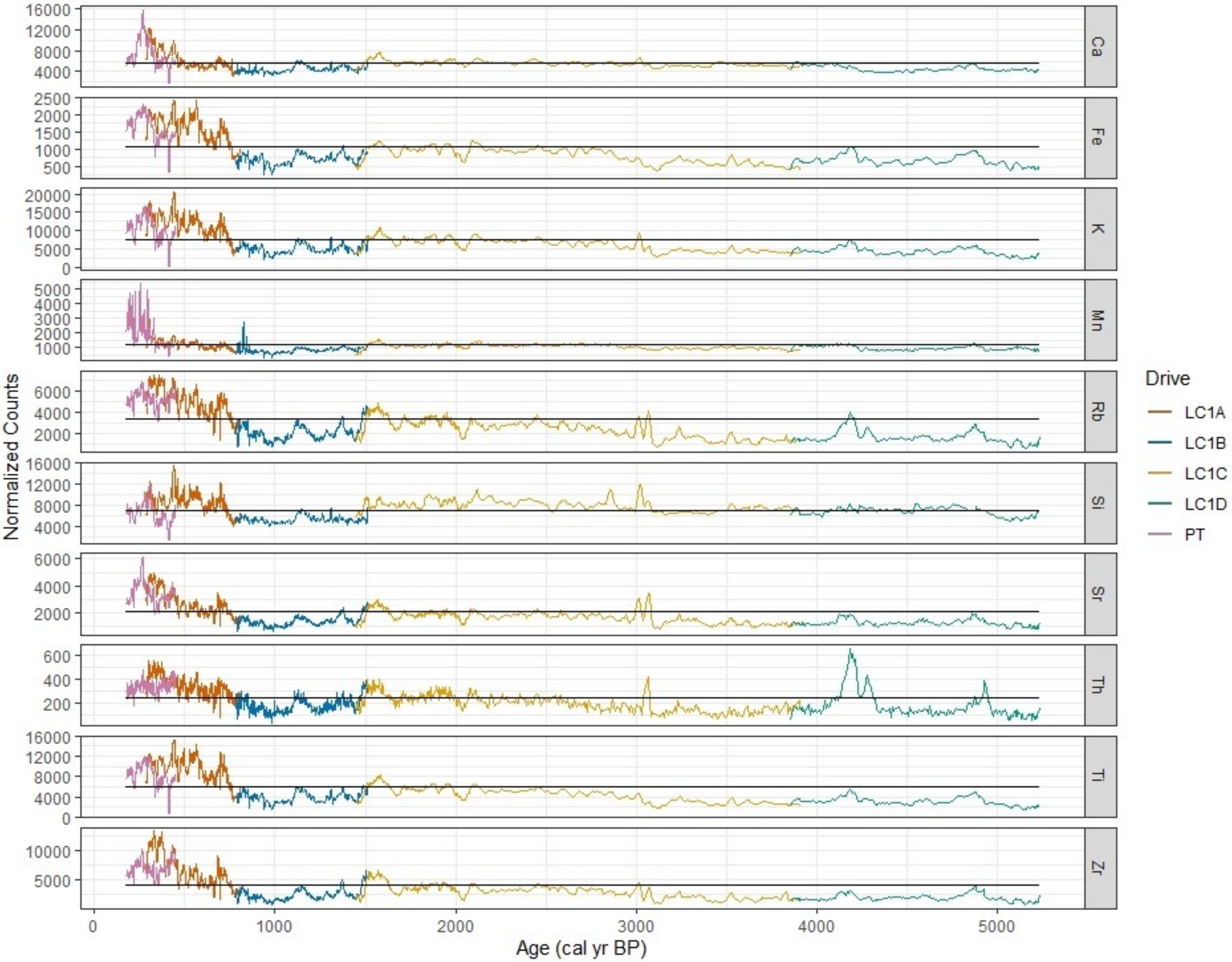

Supplementary Figure 3

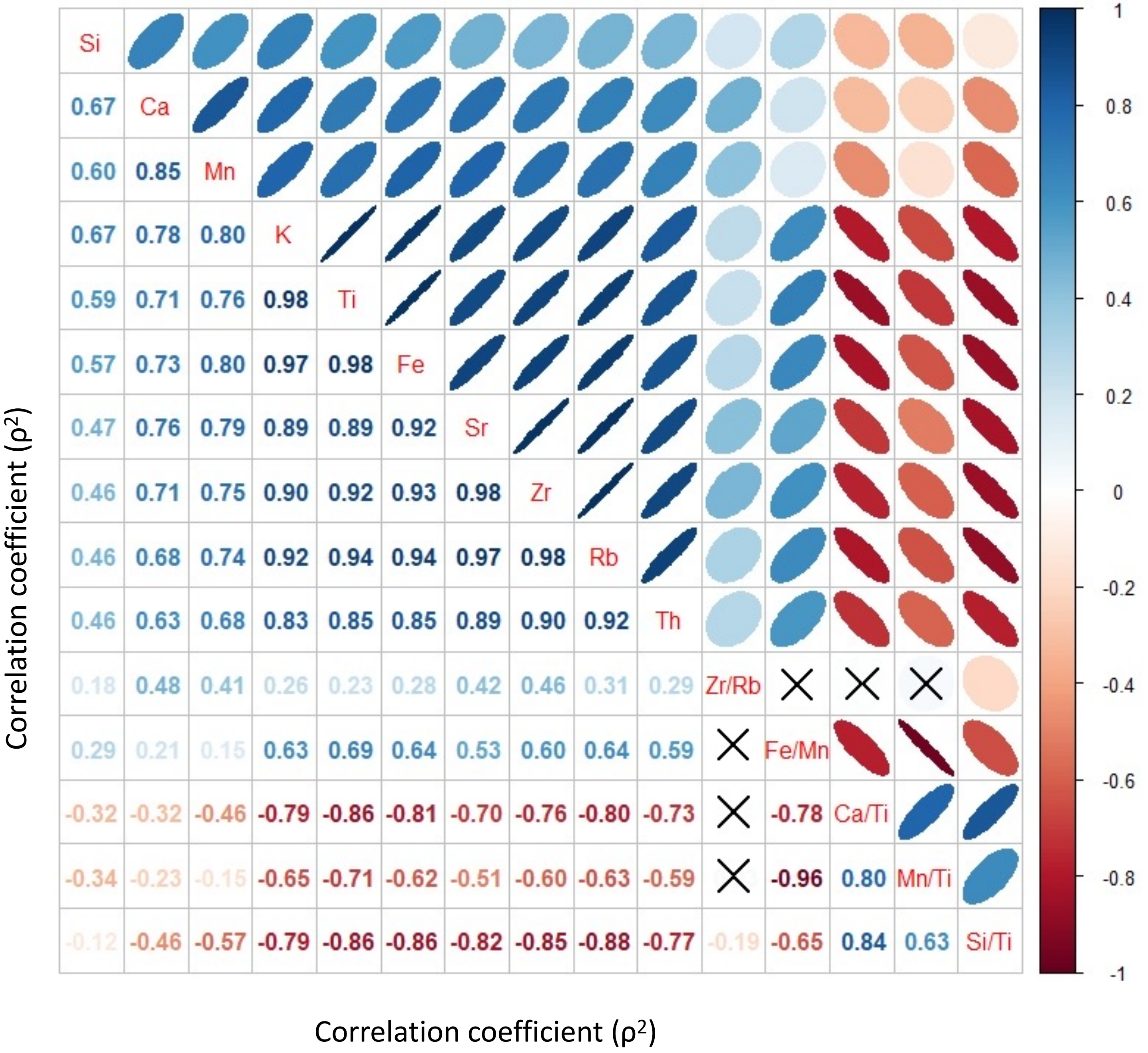

Supplementary Figure 4

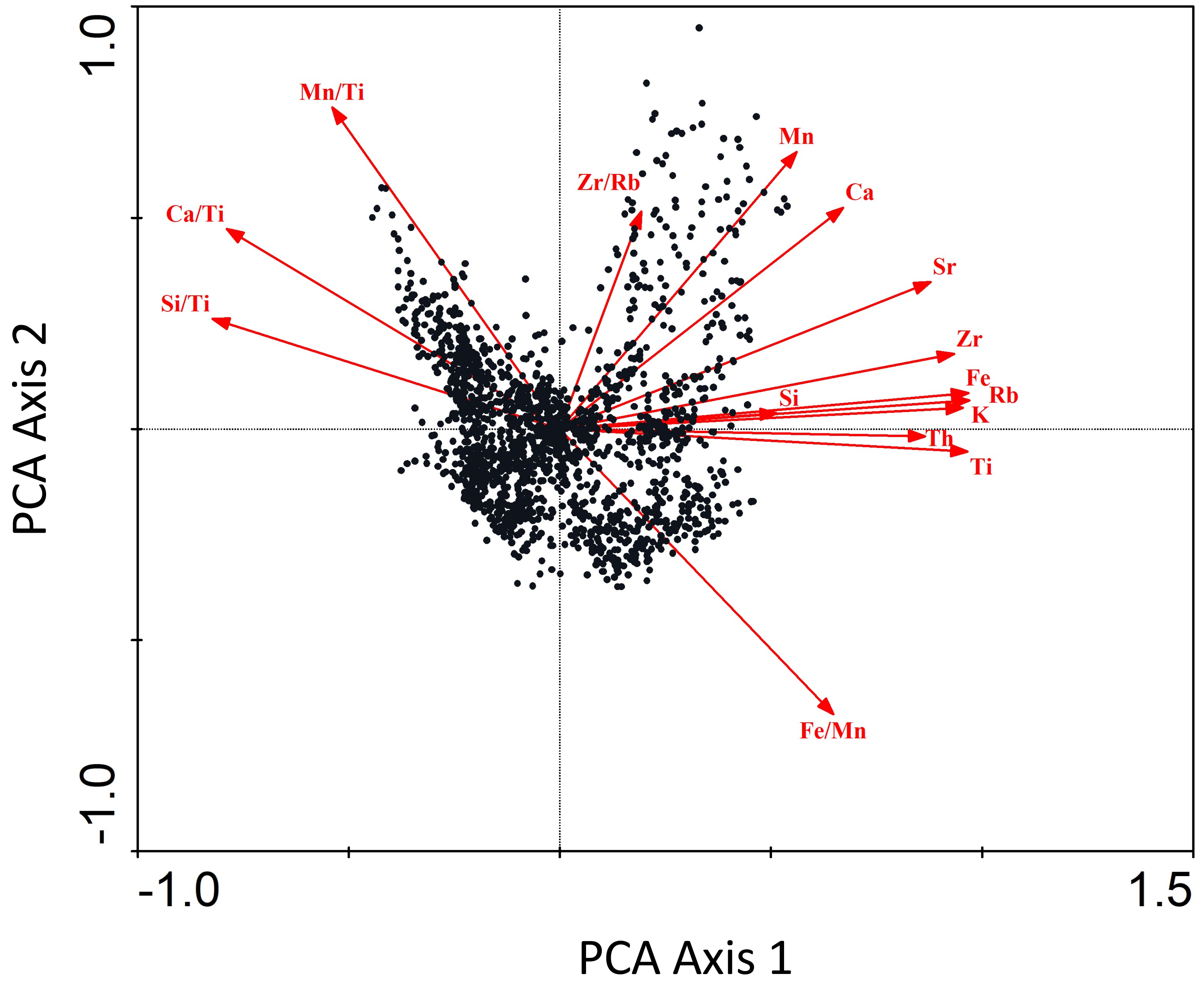

Supplementary Figure 5

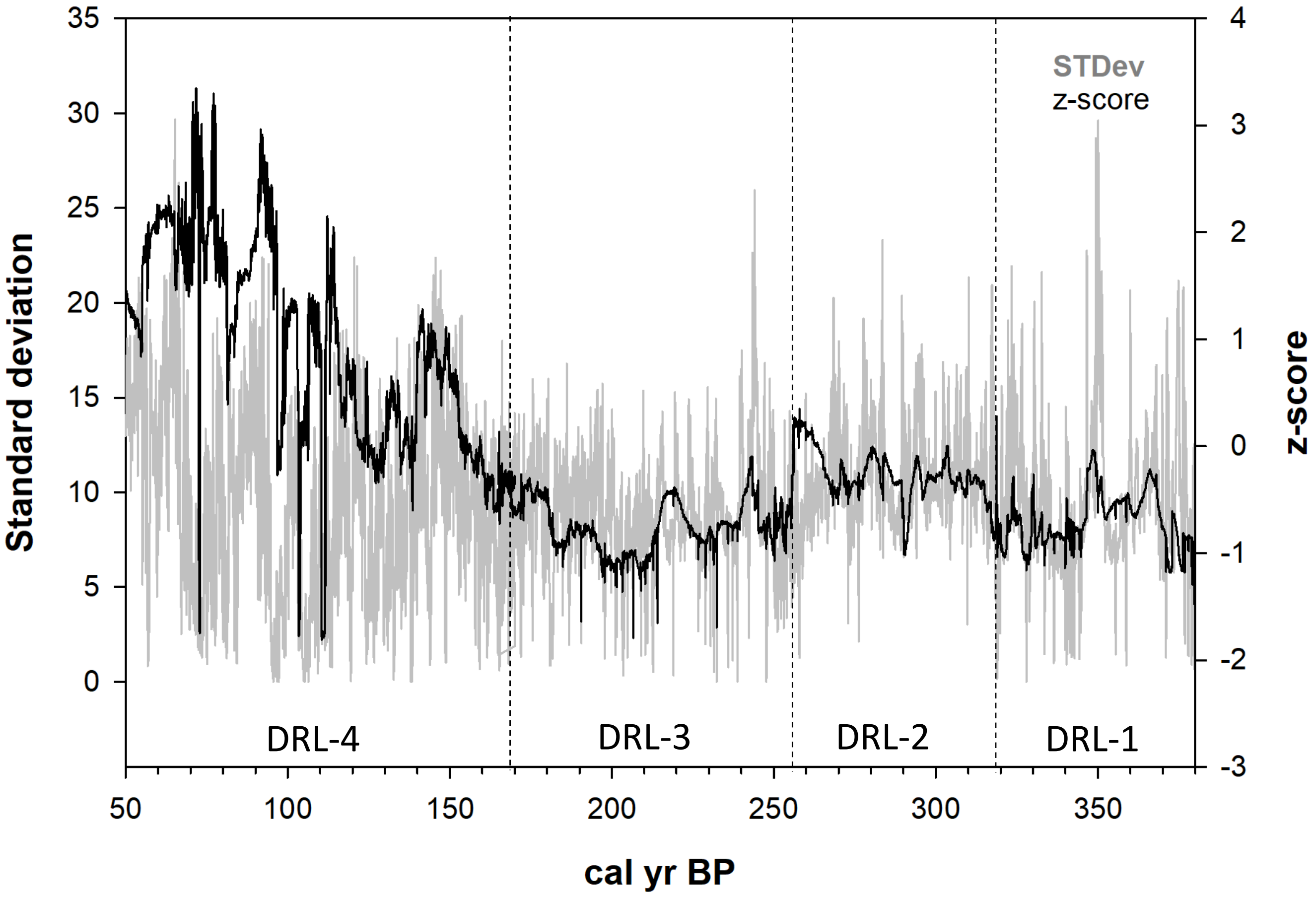

Supplementary Figure 6

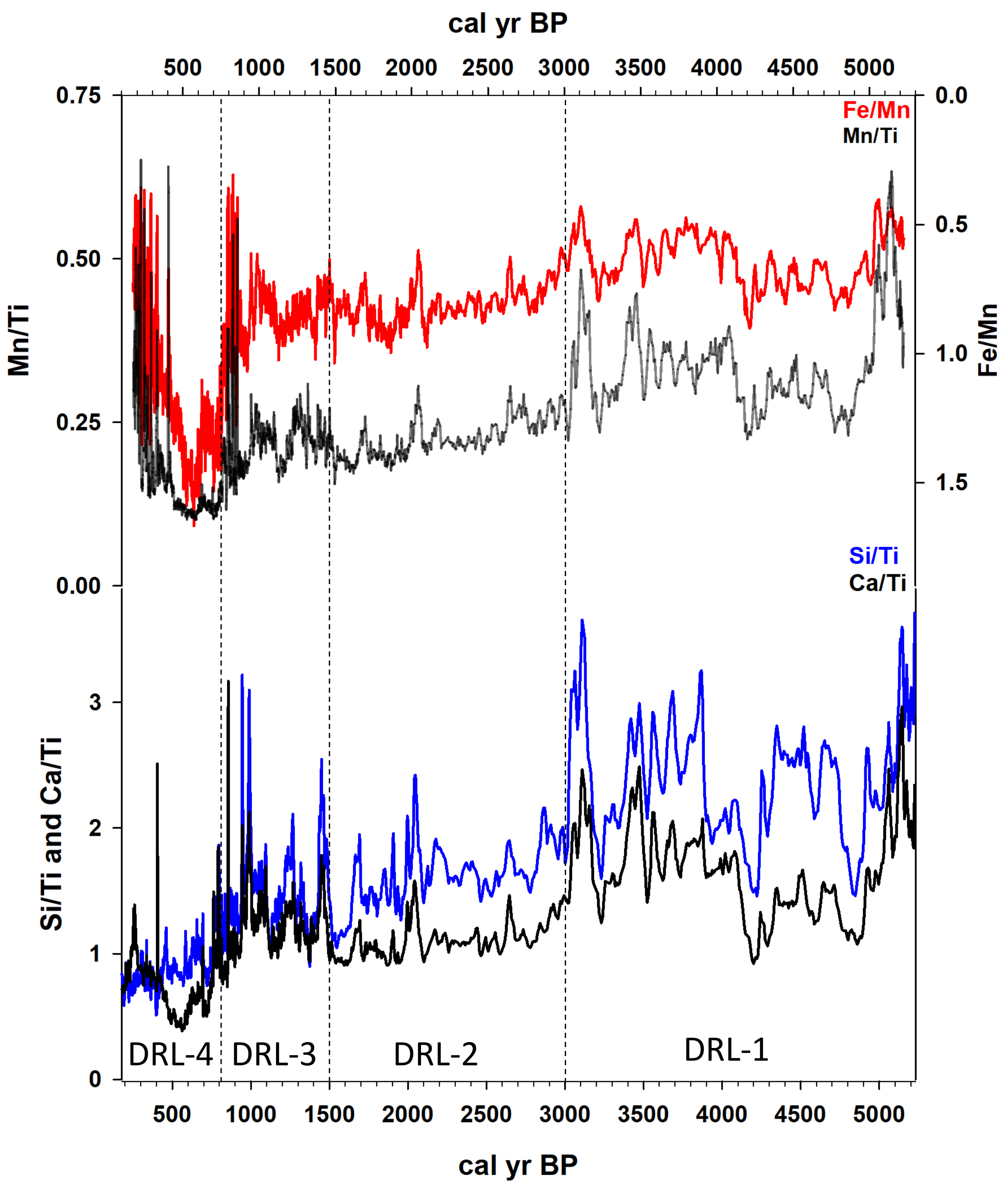

Supplementary Figure 7

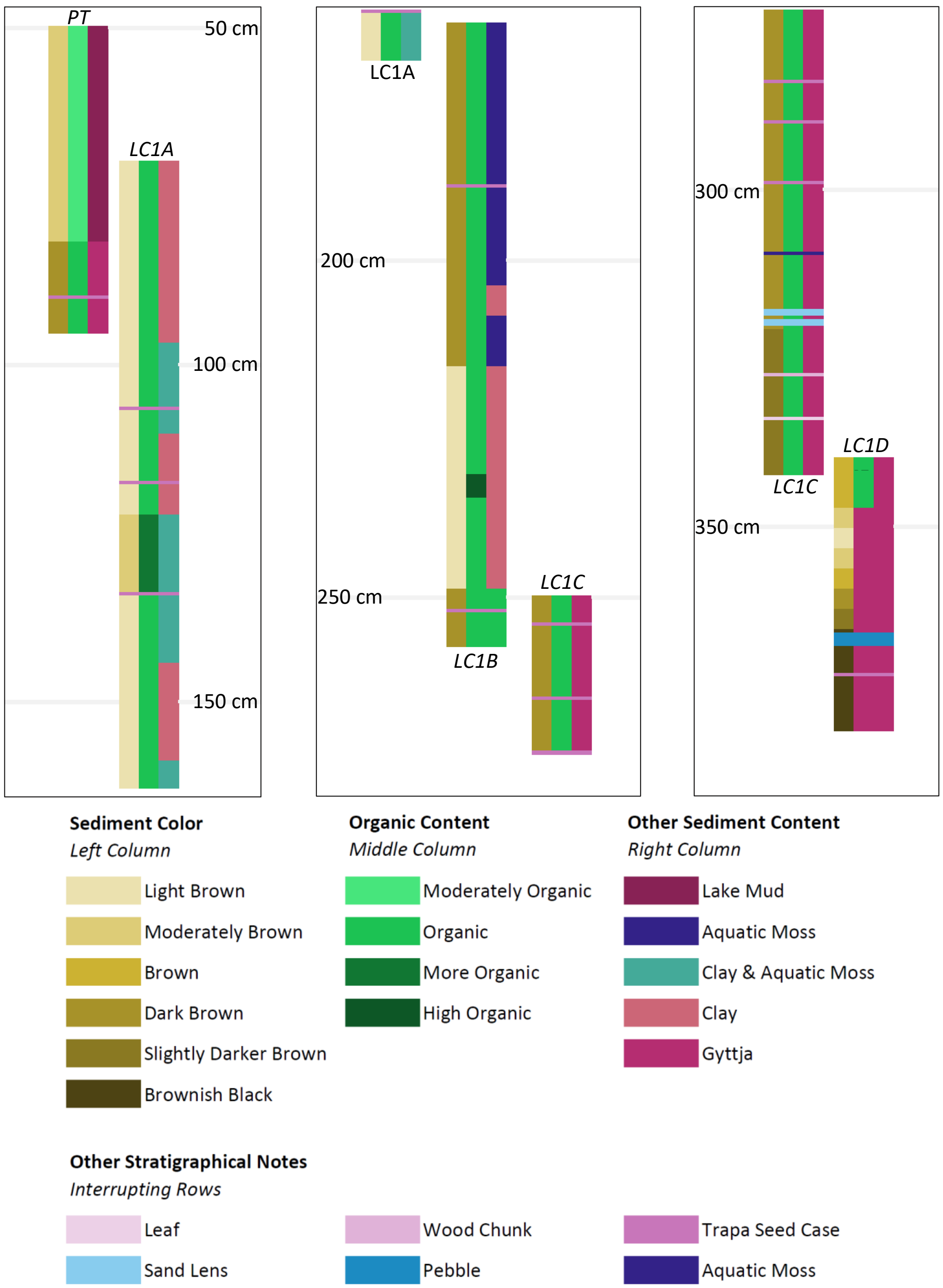

Supplemental Figure 8

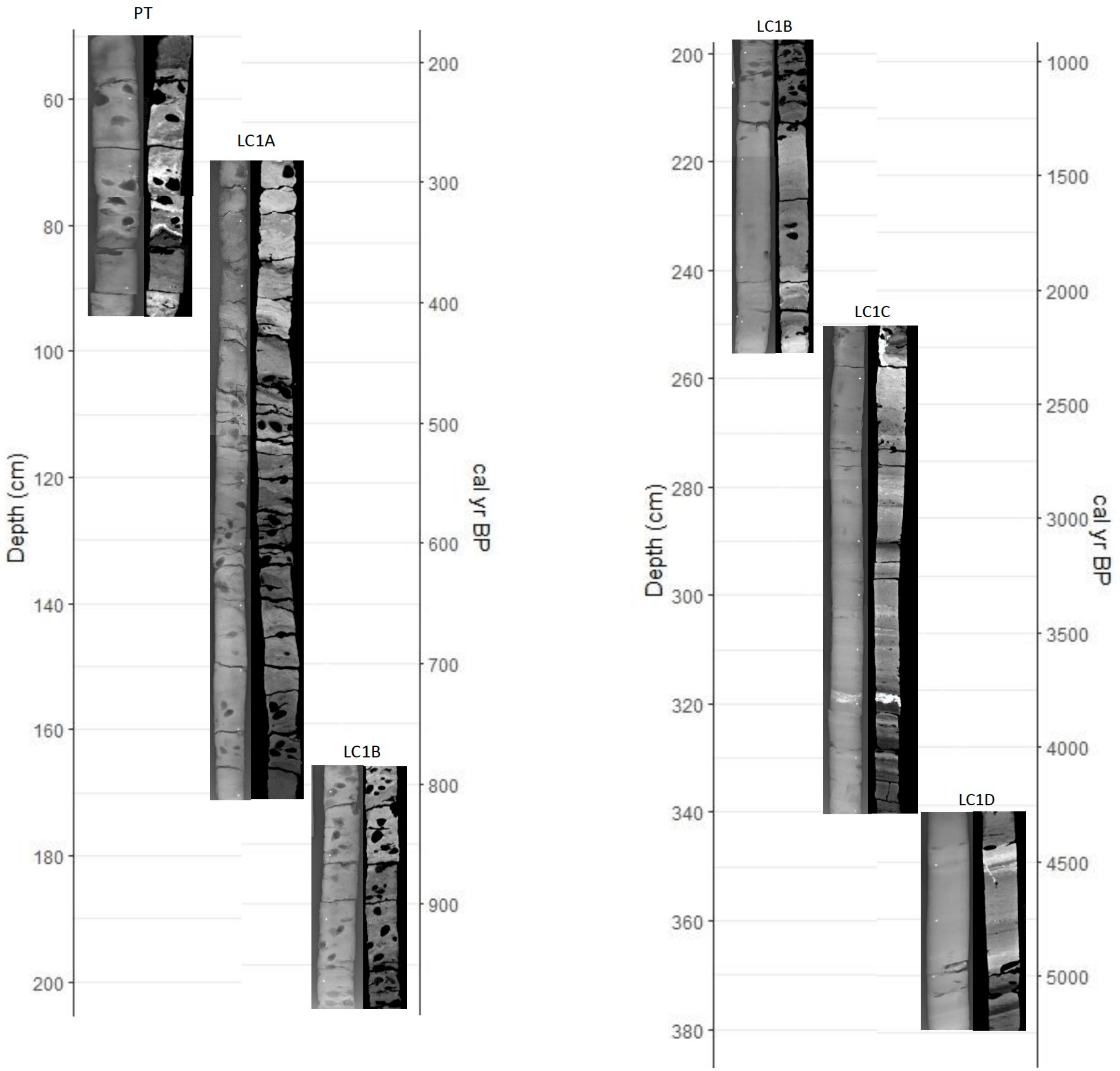

Supplementary Figure 9

Depth (m)	SWT (°C)	DO (%)	DO (mg/L)	pH
0.50	19.40	79.50	5.52	9.42
1.00	19.40	80.00	5.60	9.44
2.00	17.00	97.10	7.18	9.54
3.00	13.10	108.40	8.56	9.46
4.00	9.40	97.70	6.97	8.60
5.00	9.00	60.70	5.12	8.14
6.00	8.20	43.10	3.72	7.72
7.00	7.40	36.30	3.16	7.22
8.00	7.10	33.10	2.94	6.93
9.00	6.70	30.40	2.73	6.75
10.00	6.60	28.00	2.62	6.58
11.00	6.50	26.60	2.29	6.48
12.00	6.50	23.50	2.11	6.40
14.00	6.40	22.00	2.00	6.33
16.00	6.40	20.20	1.85	6.25

Supplementary Table S1

	Axis 1	Axis 2
**Th**	0.8656	-0.0175
**Rb**	0.9702	0.0677
**Sr**	0.8783	0.3474
**Zr**	0.9338	0.1777
**Si**	0.5137	0.0372
**K**	0.9545	0.0497
**Ca**	0.6707	0.524
**Ti**	0.9653	-0.0541
**Mn**	0.5609	0.6561
**Fe**	0.9674	0.0848
**Zr/R b**	0.1925	0.5147
**Fe/M n**	0.6482	-0.6759
**Ca/T i**	-0.7893	0.4732
**Si/T i**	-0.8229	0.2608
**Mn/T i**	-0.5392	0.761
**Proportion of Variance**	61.3	16.4
**Cumulative Variance**	61.3	77.7

Supplementary Table S2
